# Brain CT in Patients With Altered Mental Status: Tertiary Care Emergency Department Review

**DOI:** 10.7759/cureus.100504

**Published:** 2025-12-31

**Authors:** Maan Jamjoom, Faisal Boker, Abeer Alraiqi, Rahaf Organji, Mohammed Alotaibi, Abdulrahman Alghamdi, Nawaf Alharbi, Waleed Aljehani, Saleh Alqadi, Lara Aljohny, Belal Alturkistani

**Affiliations:** 1 Emergency Medicine, King Abdullah International Medical Research Center, Jeddah, SAU; 2 College of Applied Medical Sciences, King Saud Bin Abdulaziz University for Health Sciences, Jeddah, SAU; 3 Emergency Medicine, Ministry of National Guard Health Affairs, Jeddah, SAU; 4 Medicine, King Saud Bin Abdulaziz University for Health Sciences, Jeddah, SAU

**Keywords:** altered mental status, ct brain, pathology, risk, stroke

## Abstract

Background

Altered mental status (AMS) is a frequent and non-specific presentation in the Emergency Department (ED), reflecting a wide range of underlying conditions, from benign to life-threatening. Identifying patients who require urgent neuroimaging remains a clinical challenge, as overuse of CT brain scans increases costs and radiation exposure, while underuse may delay critical diagnoses. Despite its prevalence, there is no standardized approach to guide CT utilization in AMS cases, leading to inconsistent practices. This study aims to analyze CT brain findings among AMS patients who received imaging in the ED of King Abdulaziz Medical City, Jeddah, to determine its necessity.

Method

This analytical cross-sectional study was conducted in King Abdulaziz Medical City between January 2023 and December 2023. The sample of the study included all the patients who presented to the ED with AMS and had undergone a CT scan of the brain.

Results

Among 112 AMS patients who underwent CT brain imaging, 29.5% had positive findings, most commonly intracranial hemorrhage (9.8%) and stroke (7.1%). Hypertension (61.6%) and diabetes mellitus (50%) were the most prevalent comorbidities. CT positivity did not differ significantly by time of imaging (p = 0.477).

Conclusion

CT brain was positive in nearly one-third of AMS patients in this study, identifying critical conditions like hemorrhage and stroke. This underscores its necessity in high-risk patients, especially those with multiple comorbidities, focal deficits, trauma, or anticoagulant use. However, the majority of the scans showed no acute findings. We emphasize the future need for prospective studies with control groups and validated risk stratification tools to safely guide CT utilization in AMS patients.

## Introduction

Altered mental status (AMS) is a common presenting complaint in the Emergency Department (ED), reported in approximately 4% to 10% of patients [1,2]. AMS is a non-specific term describing a range of cognitive and neurological disturbances, often signaling an underlying medical condition. Early recognition and management are vital to reduce the risk of complications, prolonged hospitalization, and mortality [3]. Conditions contributing to AMS can be broadly classified into central nervous system (CNS) and non-CNS causes. CNS-related factors include stroke, seizures, and encephalitis, while non-CNS causes encompass sepsis, metabolic disturbances, cardiogenic shock, and intoxication [1]. While infections, metabolic disturbances, and toxicological causes are less common overall, they are more prevalent in younger age groups [4].

A major challenge in managing AMS patients is determining who should undergo immediate CT brain imaging. While CT scans are crucial for detecting life-threatening conditions, their overuse can lead to unnecessary radiation exposure and higher healthcare costs [5]. On the other hand, underuse may result in delayed diagnoses, potentially compromising patient outcomes and increasing the risk of severe complications. Studies have shown significant variability in the use of CT for AMS patients, often driven by individual clinician judgment rather than evidence-based guidelines [6]. The lack of standardized criteria for CT imaging in AMS underscores the need for a more structured and universally accepted decision-making process.

The root of this issue lies in the lack of unanimity on which risk factors predict abnormal CT findings in AMS patients. Some studies suggest that age, a history of trauma, and focal neurological deficits are strong predictors, but these factors are inconsistently applied in clinical practice [7]. Additionally, the wide range of potential causes of AMS makes it difficult to create universal guidelines that are applicable across diverse patient populations. This variability in clinical judgment further complicates the decision-making process, often leading to inconsistent management practices. Without a clear risk stratification tool, clinicians often rely on subjective judgment, which leads to variability in CT use and potentially less optimal patient care and outcomes [8].

A study aimed at creating a straightforward clinical score to determine the necessity of cranial CT in ED patients with suspected non-traumatic intracranial pathology. The proposed score assists clinicians in making informed decisions regarding imaging needs [9]. Applying a similar approach to AMS could improve both diagnostic efficiency and resource use in EDs.

This study aims to analyze CT brain findings among AMS patients who received imaging in the ED of King Abdulaziz Medical City, Jeddah, to determine its necessity. In addition, the study seeks to explore the impact of various clinical factors on CT decision-making, helping to minimize unnecessary imaging while ensuring timely diagnosis.

## Materials and methods

Study design

The study design used is an analytical retrospective cross-sectional study. The study was conducted in the ED at King Abdulaziz Medical City, Jeddah, Saudi Arabia, between January 2023 and December 2023.

Consent and ethical approval

The study was carried out in line with the Helsinki protocol, and ethical approval from the Institutional Review Board of King Abdullah International Medical Research Center (KAIMRC), KSAU-HS, Jeddah, was duly acquired prior to conducting this study. No participant names or identification were gathered, and the data were stored on the principal investigator’s secured system, which was difficult for unauthorized individuals to access.

Study sample

The sample size was randomized. The initial screened sample was 156. The analyzed sample size was 112 patients, the ones who registered in the ED with AMS and had a brain CT from January 2023 to December 2023, and met the inclusion criteria. 

The inclusion criteria were patients presenting to the ED with AMS who had undergone a brain CT. Exclusion criteria were patients presenting with other complaints than AMS, and patients with AMS who did not have a brain CT or had not done a brain CT later (more than one week from the time of presentation) for other reasons.

Data collection tool

Data were gathered through the review of medical records of patients who visited the ED with complaints of AMS and had a brain CT scan, who met the specified criteria. Data were collected by the authors of this study. Collected information consisted of patients' characteristics, symptoms upon arrival, comorbidities, risk factors, and findings from brain CT scans.

Data and statistical analysis

The data gathered were organized with the use of Microsoft Excel (Microsoft Corp., Redmond, WA). Information on patients who do not meet our criteria was deleted from the dataset. SPSS was used to analyze the data. In order to summarize the demographic, comorbidities, and risk factors of the participants in the study, descriptive statistics (mean and standard deviation) were used for continuous variables such as age. Categorical variables such as gender, history of head trauma, CT findings, existing comorbid conditions, and risk factors were presented in frequency and percentage. Chi-square and t-tests were used to investigate the relationship between the timing of the CT scan and the result of the CT scan. The confidence was set at 95%, and Statistical significance was determined at p ≤ 0.05. Due to the retrospective design, this study mainly describes associations.

## Results

From January 2023 to December 2023, we had a sample size of 156. We excluded 44 due to not fitting our inclusion criteria or incomplete data in Electronic Records (BestCare, Seoul, South Korea). As a result, we included 112 patients. Among the 112 patients, 59 (52.7%) were males, and 53 (47.3%) were females. The mean age of patients in years was 61.3 (±21.4). Regarding the comorbidities and risk factors, the most common one was hypertension which was 69 (61.6%) of patients, followed by diabetes mellitus (56, 50%), past stroke (28, 25%), neurological disease (28, 25%), epilepsy (18, 16.1%), ischemic heart disease (19, 17%), dyslipidemia (27, 24.1%), cancer (17, 15.2%), metastatic cancer (11, 9.8%), anticoagulation use (14, 12.5%), recent head trauma (16, 14.3%), heart failure (10, 8.9%), and smoking (three, 2.7%), as illustrated in Table 1.

**Table 1:** Bio-demographic characteristics and risk factors

Bio-demographic data	Frequency (%), mean (±SD) (n = 112)
Age in years	61.3 (±21.4)
Gender	
Male	59 (52.7%)
Female	53 (47.3%)
Comorbidities and risk factors	
Hypertension	69 (61.6%)
Diabetes mellitus	56 (50%)
Past stroke	28 (25%)
Neurological disease	28 (25%)
Epilepsy	18 (16.1%)
Ischemic heart disease	19 (17%)
Dyslipidemia	27 (24.1%)
Cancer	17 (15.2%)
Metastatic cancer	11 (9.8%)
Anticoagulation use	14 (12.5%)
Recent head trauma	16 (14.3%)
Heart failure	10 (8.9%)
Smoking	3 (2.7%)

Positive CT findings were noticed in 33 (29.5%) of patients, the most common CT finding was intracranial hemorrhage (11, 9.8%), followed by stroke (eight, 7.1%), brain lesions (4, 3.6%), sinus changes (three, 2.7%), hydrocephalus (two, 1.8%), meningioma (two, 1.8%), as illustrated in Table 2.

**Table 2:** CT findings

CT finding	Frequency (%)
Any CT finding	33 (29.5%)
Intracranial hemorrhage	11 (9.8%)
Stroke	8 (7.1%)
Brain lesions	4 (3.6%)
Sinus changes	3 (2.7%)
Hydrocephalus	2 (1.8%)
Meningioma	2 (1.8%)

Regarding the CT scans by time of performance and presence of finding, we found that the majority of patients came at the evening time (41, 36.6%) and had the most positive findings with 14 (34.1%) positive scans and 27 (65.9%) with negative findings, coming after in frequency is the night time (40, 35.7%) with (nine, 22.5%) positive scan and (31, 77.5%) with negative scans, and day time with 31 (27.7%), with 10 (32.3%) positive scans and 21 (67.7%) negative scans. There was no statistically significant association between the presence of CT findings and the time of obtaining the image; χ²(2, N = 112) = 1.48, p = 0.477, with Cramér’s V = 0.115, indicating a small effect size (Table 3).

**Table 3:** CT scans by time of performance vs. presence of finding *χ²(2, N = 112) = 1.48, p = 0.477. Cramér’s V = 0.115 (small effect size)

Timing of image	Frequency (%)	Findings present		p-value
		Positive scan	Negative scan	
Daytime	31 (27.7%)	10 (32.3%)	21 (67.7%)	0.477*
Evening time	41 (36.6%)	14 (34.1%)	27 (65.9%)	
Nighttime	40 (35.7%)	9 (22.5%)	31 (77.5%)	

## Discussion

This study examined the spectrum of CT brain findings, risk factors, and comorbidity in patients presenting to the ED with AMS. A total of 112 patients presented to the ED with AMS, with a total number of 59 (52.7%) male and 53 (47.3%) female. Leong et al. showed a slightly different gender distribution, with 48% male and 52% female [10]. Among the 112 patients, 33 (29.5%) showed positive CT findings. Similarly, Leong et al. showed 36% of abnormal CT scans [10]. Hypertension was the commonest comorbid in our sample, representing 61.6%; however, Kumari et al. [11] reported 45.2% patients with hypertension. What might explain this is that our population's diet usually contains high salt, and they have poor physical activity. Diabetes mellitus was represented in half of our sample (50%). On the other hand, diabetes mellitus represents only 9.5% in Kumari et al.’s paper [11]. This could be explained by the sedentary lifestyle and high-glycemic-index foods that our population is eating.

The most common brain pathology reported was intracranial hemorrhage (11, 9.8%). However, another study by Leong et al. reported 34.4% in the age group 18-64 and 20.6% in the age group above 65 [12]. This variation can be cleared by the fact that this study has a small sample size compared to others. Why were CT scans negative in 70.5%? It can be answered as the AMS might be caused by underlying metabolic, toxic, or systemic conditions that are not detected by CT scans, requiring emergency physicians to explore the variety of causes [5]. Acharya et al. found that the use of non-contrast CT head in patients with AMS is high, with a low likelihood of detecting significant abnormalities [9].

The association between the patient's comorbidity and risk factors and CT result, which we seek to contribute to a broader understanding of the correlation between the two. It is important to mention that 11 patients presented with intracranial hemorrhage were not on anticoagulant therapy, and seven of the 11 had no head trauma. This observation underscores the importance of exploring other potential risk factors that may contribute to this clinical presentation. It may be due to hypertensive hemorrhage, which is a complication of chronic hypertension. Another possible etiology can be amyloid angiopathy, particularly in elderly patients. Further risk factor identification is needed.

The statistical analysis revealed no association between the timing of the CT scan and positive results, as seen in Table 3 (p = 0.477). Nighttime showed the most negative results, reaching 77.5%. The overflow of patients during nighttime, particularly in the ED, may have influenced the clinical decision-making. However, this leads to delaying scans for patients who might have benefited from earlier imaging and exposing those who do not need it to avoidable radiation. These observations clarify the need for further research into the interplay between ED quality, physician decision-making, and the usage of CT in cases of AMS.

A recent systematic review and meta-analysis showed that 96% of patients who present to the ER with AMS get a CT of the head, with a 17% rate of positive CT findings [8]. This raises the question of CT overuse in the ED, which may cause unnecessary radiation exposure and increased financial costs on the health care system [13].

The American Academy of Family Physicians recommends that for patients without an obvious cause of AMS, a non-contrast head CT scan is appropriate [14]. Nowadays, physicians tend to practice defensive medicine to avoid any legal responsibilities [15,16]. This is also eased by the wide availability of CT scans and the rapid results [16]. In 2004, Lee et al. reported that 91% of ER physicians believed there is no increased risk of cancer when ordering a CT scan [17]. Another aspect of the problem is the lack of clear clinical guidelines or a risk stratification tool that can confidently classify patients who get a CT scan and who do not. In 2015, Bent et al. suggested a simple algorithm that can help stratify patients based on the presence of risk factors. The most significant risk factors were the presence of a focal neurological deficit and age above 55 [18]. Additionally, a newer algorithm was suggested to help classify patients presenting with AMS [19]. Although many efforts have been put into developing a risk stratification tool, there is not a widely accepted and verified one yet.

Limitations

The sample size in this study was smaller compared to others. Also, it is a single-center study. Moreover, due to the lack of total ER visit numbers in the study period, the comparison between the rate of using CT scans in patients presenting with AMS and those with other complaints was not feasible. The presence of different outcome assessors and the lack of a standardized CT interpretation approach can have an impact on the results.

## Conclusions

CT brain was positive in nearly one-third of AMS patients in this study, identifying critical conditions like hemorrhage and stroke. This underscores the necessity of CT in high-risk patients, especially those with multiple comorbidities, focal deficits, trauma, or anticoagulant use. However, with the majority of the scans showing no acute findings, there is a need for a reliable risk stratification tool. We emphasize the need for future prospective studies with control groups and validated risk stratification tools to safely guide CT utilization in AMS patients.
